# Five meaningful roles of engagement: Framework for Lived Experience Roles in Mental Health Research (FLER-R)

**DOI:** 10.1371/journal.pmen.0000632

**Published:** 2026-08-21

**Authors:** Annelin Festervoll, Ernesto Isaac Lara, Conor Gavin, Novembre Mercier, Michael J. Norton, Helene Speyer

**Affiliations:** 1 TIPS – Clinical Research in Psychosis, Stavanger University Hospital, Stavanger, Norway; 2 Department of Global Health and Social Medicine, Harvard Medical School, Boston, Massachusetts, United States of America; 3 Department of Health Policy and Management, University of California, Los Angeles, California, United States of America; 4 St John of God Research Foundation, St John of God Hospitaller Services Group, Dublin, Ireland; 5 CRISP – Comprehensive Research into Schizophrenia and other Psychopathologies, Douglas Mental Health University Institute, Montreal, Canada; 6 UCD School of Medicine, University College Dublin, Dublin, Ireland; 7 CORE – Copenhagen Research Center for Mental Health, Mental Health Center Copenhagen, Mental Health Services in the Capital Region of Denmark, Copenhagen, Denmark; 8 Department of Clinical Medicine, Faculty of Health Sciences, University of Copenhagen, Copenhagen, Denmark; PLOS: Public Library of Science, UNITED KINGDOM OF GREAT BRITAIN AND NORTHERN IRELAND

## Abstract

People with lived experience are increasingly recognized as essential contributors to mental health research. Although a growing body of literature, including frameworks and guidance documents, address lived experience involvement, no existing work has systematically identified and defined the distinct roles through which people with lived experience engage in mental health research. This conceptual essay combines a narrative review of peer-reviewed and grey literature with the collective practice-based experience of the author team as lived experience contributors. The Framework for Lived Experience Roles in Mental Health Research (FLER-R) was iteratively developed and refined through collaborative consensus-building among the authors. FLER-R delineate and describe five roles which people with lived experience may undertake in mental health research, supporting the structural embedment of lived experience across mental health research practices.

## Lived experience in mental health research

Lived experience (LE), sometimes referred to as lived expertise, in mental health research is in this paper defined as first-hand experiential knowledge of living with mental health challenges, and/or navigating the systems of care aimed at promoting recovery for mental health challenges. Rather than being solely defined by single personal narratives, lived experience knowledge is shaped by historical and structural dynamics.

The broader Lived Experience movement has a long and multifaceted history, existing within a wider context of transnational, political and societal trends [1–4]. It is rooted in collective liberation, health agency and justice. LE in mental health research spans various fields, including youth mental health [5–7], global mental health [8–10], public health [10], stigma and discrimination [11] and psychosis studies [2,12]. LE advocacy and activism have for decades played a central and necessary role in driving positive developments in mental health services, policy and research. However, the field is seemingly becoming fragmented, with diverging perspectives and contested understandings of what lived experience engagement is and should entail.

Concurrently, a growing body of work has emerged on the inclusion of lived experience in mental health research, with Greenhalgh et al. [13] identifying 65 published examples. These fall broadly into five categories: power‐focused; priority‐setting; study‐focused; report‐focused; and partnership‐focused. Existing contributions in the broader landscape includes long-standing traditions of consumer and survivor-led research [14] and mad studies [15], philosophical and conceptual perspectives [16], commentaries [17], and original empirical research [18]. Despite the multiple attempts to conceptualize and theorize lived experience involvement, the literature remains fragmented—using partially overlapping terms such as service user/patient involvement, lived experience involvement, or patient and public involvement. Challenges continue to persist [19,20], including questions of legitimacy, epistemic authority, and the practical difficulties of peer reviewing lived experience-based manuscripts [14,21].

The oldest and most cited are power-focused frameworks. Arnstein’s Ladder of Citizen Participation [22] and its derivatives, including the IAP2 Public Participation Spectrum [23] and Hart’s Ladder [24], describe involvement as a continuum from tokenism to citizen control. These frameworks have been invaluable for identifying when involvement is superficial, but they measure degree of influence rather than defining the roles through which involvement occurs.

Process and standards frameworks address a different question: how involvement should be conducted. The 4Pi National Involvement Standards [25] co-produced by mental health service users, articulates five domains of good practice—Principles, Purpose, Presence, Process, Impact—underpinned by Power. The GRIPP2 checklist [26] aims at standardizing reporting, while the WHO Framework for Meaningful Engagement [27] establishes global principles around dignity, equity, and the transfer of power to people with lived experience (PWLE). Each framework makes a genuine contribution, yet none defines the roles through which these principles are enacted.

A smaller number of frameworks have moved toward role differentiation. The Involvement Matrix [28] names five roles—Listener, Co-thinker, Advisor, Partner, Decision-maker—but functions as a project-level negotiation tool, not an institutional typology, and was not developed for mental health contexts. Suomi et al.‘s Framework for the Engagement of People with a Lived Experience in Program Implementation and Research [29] addresses engagement across research and services broadly, but without defining distinct roles or the obligations each entail. Sacred Heart Mission’s Lived Experience Advisory Framework [30] delineates five levels of participant voice, but was designed for a homelessness services organization rather than research institutions. Preberg and colleagues [7] describe engagement levels in youth mental health research, and Gupta and colleagues [18] map LE researcher identity—both valuable, but neither addresses the training, governance, or institutional obligations each role requires.

No existing framework has systematically named and defined the distinct positions through which PWLE engage in mental health research. This absence has concrete consequences. Without named roles, there is no basis for the obligations that should follow. Institutions cannot be held accountable for training they were never required to provide. PWLE cannot claim compensation that was never specified. The result is a field where involvement is simultaneously mandated and structurally unsupported. Named roles generate obligations: to train, to compensate, to support. FLER-R provides this typology, to the best of our knowledge, for the first time.

## The framework for lived experience roles in mental health research (FLER-R)

In this framework, we outline five distinct roles through which PWLE may be engaged with in mental health research, broadly they are defined as: participation; consultancy; partnership; governance - administrative; and governance - research (see Table 1).

**Table 1:** The five meaningful roles in FLER-R.

Type	Description	Example(s) of Activities
***Participation: Research Volunteer***	PWLE contribute their experiential knowledge primarily as participants within research studies. Perspectives are treated as valuable data and do not require formal research training to take part in.	• Participating in focus group discussions, interviews, or surveys • Testing and providing feedback on intervention materials
***Consultantcy: Advisory Board Member***	PWLE provide structured, advisory input on research priorities, design, processes, and interpretation through advisory board roles or formal consulting roles.	• Serving on expert advisory boards or committees • Reviewing grant proposals, study protocols, recruitment strategies, and/or outcome measures • Advising on interpreting data and ethical considerations
***Partnership: Peer Researcher***	PWLE function as an integrated member of the research team, contributing across multiple stages of the research cycle and work being tied to specific research deliverables.	• Co-developing research questions, study designs, inclusion criteria, etc. • Supporting recruitment and outreach efforts, and data collection, analysis, and interpretation • Co-authoring dissemination efforts such as publications and presentations
***Governance: Administrative Leader***	PWLE hold formal administrative leadership roles, such as “Head of Lived Experience” to strategically lead organization efforts to strategically embed lived experience involvement within organizational activities.	• Curating, forming, and leading lived experience programs • Supervising and mentoring PWLE throughout the research process • Developing institutional level policies, trainings, and partnerships to enhance lived experience engagement
***Governance: LE Researcher***	PWLE act as a researcher with independent responsibilities equivalent to traditional researcher. They are grounded in a lived experience informed perspective.	• Leading, co-leading or contributing to research projects, including contributing as co-applicants on funding proposals • Decision-making authority, including budgetary autonomy • Contributing to the advancement of research fields by challenging assumptions and shaping research agendas, sometimes within or alongside traditional academic roles

First, the authors conducted a narrative review of the existing literature on lived experience involvement in mental health research, engaging with published frameworks, empirical studies, commentaries, and grey literature relevant to the roles, experiences, and challenges of PWLE in research settings. Second, the framework drew on the collective practice experience of the author team, who collectively hold experience across lived experience research roles in Norway, Ireland, the United States, and Canada—including as peer researchers, advisory board members, governance leaders, and independent researchers. Third, the framework was informed by sustained engagement with the broader lived experience community through professional networks, advisory roles, and community consultations. The five roles were iteratively developed, discussed, and refined across the author team until consensus was reached.

The roles differ in scope, responsibility, training requirements, years of experience, compensation, and the level of institutional support needed. These roles are not mutually exclusive, and individuals may occupy more than one role simultaneously. While the roles are not intended to be hierarchical in value (i.e., one role is “better” than the other), they reflect an increasing level of authority and research autonomy. Taken together, they also outline a potential career trajectory for PWLE in mental health research roles.

### Participation: The research volunteer

In this role, PWLE contribute to research primarily as participants. Individuals in this role can be named many things: participant; people with lived experience; community members; co-constructors and so forth. They mainly attend co-production workshops where the main goal is to “give away” information. The participant does not have any research-based autonomy in their role, but is rather responsive to the researcher. Oftentimes, and ideally in practice, PWLE are positioned as consultants or collaborators, however, this entry-level role does not require prior research training, affiliation with organizations, and/or symptom stability beyond what the individual personally deems as manageable. This has its advantages for researchers, as it requires less time, training and funding than it would to establish an advisory board or hire a peer researcher.

Although it might be less time consuming, researchers still have an ethical responsibility to provide adequate safeguards that protect PWLE during participation, including support (especially if the research has a risk of harm); clear protocols for responding to distress or activating topics; and ensure participants are provided an ethically defensible and non-coercive environment. Done correctly, evidence from LE involvement in service design suggest that involvement cultivates personal positive outcomes for PWLE [31].

A key strength of including PWLE within this role is the non-professionalized perspectives that it naturally cultivates. While professionalization of lived experience roles can bring legitimacy and advance mental health equity, there is also concerns and risks of over-professionalization [32] and commodification [33]. Participant-approaches help counteract these risks by emphasizing expertise that is less shaped by institutional practices, dominant paradigms (e.g., biomedical model), and academic incentives.

We view that including PWLE exclusively in this role only, is not in-line with the current developments in mental health research. Involving people in this role only should not be regarded as adequate lived experience involvement. However, we do acknowledge that this role is essential to data collection, but would recommend additional engagement in one or more of the other roles presented in this framework.

### Consultation: The advisory board member

As an advisor or consultant in an advisory board, PWLE provide structured input on research priorities, design, and interpretation often without being an embedded member of the research team. The advisory boards may have many different names and abbreviations, e.g., Lived Experience Advisory Board (LEAB), Lived Experience Advisory Panel (LEAP), Lived Experience Panel (LEP), Lived Experience Steering Committee, ect.. An advisory board is structurally embedded either on project level (e.g., accessible for project-members) or at the institutional level (e.g., accessible to the research group/lab, academic institution and/or hospital conducting clinical research).

An advisory board member typically require some foundational training, such as basic research knowledge and an understanding of how experiential knowledge may inform and/or improve the research project. Most PWLE enter these roles through user-led organizations and may be linked to mental health advocacy or activism. Notably, political opinions and underlying agenda’s related to organizations may be mixed with the individuals own lived experience perspectives, thus awareness and transparency may be important for all parts.

Symptom stability is not needed but being able to communicate needs, handle possibly challenging topics and being able to understand the role and the corresponding expectations is important. Here, project coordinators or institution administrators have a responsibility to thoughtfully organize institutional support and information flow for members of the advisory board.

### Partnership: The peer researcher

A peer researcher are individuals with lived experience invited to take part in the research project, not as participants, but as co-researchers under guidance [34]. At this more advanced level the individuals need more specified research knowledge and/or training. Trainings could be offered by the institution itself (e.g., in-house courses and training programs), user-led organizations (e.g., trainings and courses on participatory research or communicating lived experience in research) or through formal educational tracks (e.g., college or university-level participatory research courses or introduction courses to clinical research).

Tasks may include giving input to recruitment strategies, interview questions and guides, interview participants, participate in qualitative analysis, interpreting both qualitative and quantitative results and take part in the dissemination of findings.

Being more experienced in LE service delivery is needed along with increased research knowledge. At this stage the individual is not only participating or advising on the research, they are an integrated part of the research team. As an integrated part of the team, the peer researcher should have a clearly defined role description at their workspace, including locally adapted workplace rights. They need to be trained in research skills, including how to convey the lived experience expertise in research. Naturally the role comes with increased responsibility and autonomy, which should be reflected in the role description of their workplace-contract.

### Governance: LE Administrative leader

In this role, PWLE are hired at a research lab, group or institution and lead the lived experience workforce. Often, they are given the title “Head of Lived Experience”, if they have a significant leadership role overseeing the institutions LE workforce, or “Lived Experience Coordinator”, if their leadership role is restricted within a research group or research project. Research related tasks may include coordinating workshops or advisory boards, and overseeing and ensuring progress, support and training for peer researchers. Additional work may include keeping in contact with user-led organizations to support recruitment and dissemination of research in collaboration with them.

Individuals at this level needs increased research knowledge, leadership skills and, some prior work experience in other LE service delivery roles or other related roles, before entering a administrative leader role. Which, by the nature of the inherit tasks, entails significant amounts of autonomy, independence and responsibility.

### Governance: Lived experience researcher

After years of research experience, PWLE may transition into the role of leading research. These individuals have gained enough research momentum, knowledge and skills to take on independent research tasks equal to any other researcher. They may be co-applicants on research proposals and hold grants together with team members.

As we see it, the LE Researcher role does not require to undertake the same institutional education as that of a traditional researcher (i.e., undertake a master’s or doctoral degree). Over time accumulated research experience and knowledge is enough to be recognized as a researcher who engage self-sufficiently in research-based tasks. As such substantial research experience acquired through lived experience research practice may be considered equivalent to that of individuals with traditional academic qualifications. In essence, the LE researcher explicitly integrates lived expertise into their work, thereby creating opportunities for the next generation of PWLE to learn, develop and meaningfully contribute to research.

## FLER-R as an institutional framework

As this framework illustrates, there are multiple ways to incorporate lived experience into mental health research. A persistent challenge remains the genuine and sustained integration of lived experience perspectives within research practice. Many researchers continue to express hesitation toward this shift, which may, in part, reflect limited understanding of the purpose and potential contributions of lived experience involvement.

One way to conceptualize lived experience involvement is to draw an analogy with making a methodological decision. Similar to the research question guiding the selection of appropriate methods, the type of lived experience involvement needed in a project should be determined by the research question. In this view, lived experience is not a uniform or mandatory add-on, but a form of expertise that should be strategically and thoughtfully engaged where it is both relevant and informative. Notably, this approach already assumes the presence of an established lived experience workforce within the research group, or institution, enabling involvement to be a natural part of any project rather than awkwardly appended to the research process.

Thus, embedding lived experience in research occurs across multiple, interconnected organizational levels. One level involves integrating LE perspectives into research methodologies, while a second the infrastructure of the individual research project. Most literature is currently exploring this level of involvement. Thirdly, an equally important level concerns the institutional embedding of lived experience within mental health research organizations, including governance, employment structures, and research culture, with the organizational level overseeing the projects.

**Fig 1:**
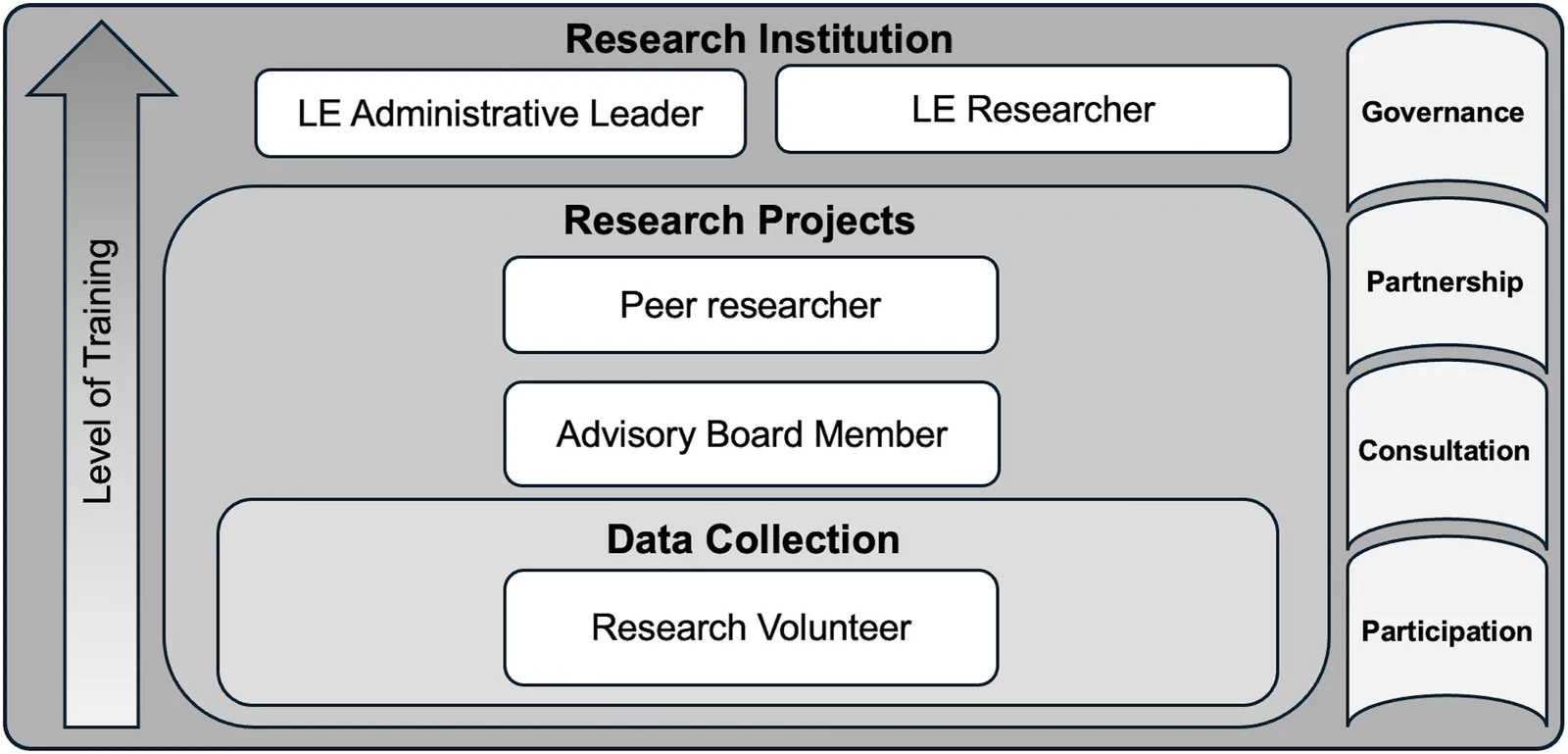
Framework for Lived Experience Roles in Mental Health Research (FLER-R). Note. The figure demonstrates how the five roles for people with lived experience may structurally be embedded within different organizational levels.

When lived experience is meaningfully integrated across all stages of the research process—data collection, project design and implementation, and institutional structures—deciding what type of involvement might be meaningful considering the research question would naturally follow and be more easily executed. Fig 1 provides an overview of the different organizational levels (i.e., institutional, project-level, and data collection–level involvement) where each type of LE research role fits.

Importantly, FLER-R was developed to delineate roles through which PWLE—without traditional research education—engage in mental health research as an explicit dimension of their institutional contribution. The framework does not, however, fully address dual-positioned researchers — individuals who combine professional research expertise with their own lived experience of mental health challenges. As Hunt [35] argue, such individuals occupy a distinct epistemic space, navigating simultaneous professional and personal identities that neither traditional researcher frameworks nor lived experience frameworks adequately capture. The distinction matters because FLER-R is concerned with how the involvement of lived experience is made accountable within research institutions. Future development of the framework should engage directly with dual positionality, and we recommend that institutions applying FLER-R develop explicit policies on disclosure and support for dual-positioned researchers, recognizing that their needs and entitlements may differ from those in dedicated LE roles.

## Risks and limitations

Clear role definitions are a necessary steppingstone towards structurally integrating experiential knowledge in mental health research but are not a sufficient condition for meaningful lived experience involvement alone. Even within well-structured frameworks, inherent tensions remain that must be actively addressed if the combination of experiential and academic knowledge is to reach its full potential. One persistent risk is tokenistic inclusion — the recruitment of PWLE into named roles without the genuine agency, resources, or influence those roles should carry. As Veldmeijer and colleagues [36] demonstrate, even design-oriented approaches that explicitly involve service users frequently fail to translate participation into real epistemic influence. Thus, without clear alignment between roles, responsibilities, and decision-making power, involvement may be diminished to ad hoc approaches increasing the risk of *involuntary tokenism* – tokenistic practice that is unintended, a result of lack of shared understanding, or a “golden standard”, of LE engagement. In other words, institutional limitations such as fast-paced research environments, resource constraints, hierarchy-culture, and unclear role definitions may impact the ability to provide environments for PWLE to influence research as their roles are intended to do.

A related and deeper challenge concerns power imbalances in how experiential knowledge is received. Speyer, Lysaker and Roe [37] term epistemic deference—a well-intentioned but ultimately harmful tendency to treat personal narratives with reverence rather than critical engagement, leaving lived experience knowledge socially acknowledged but intellectually sidelined.

As such, despite a growing recognition of LE engagement as a valuable contribution in mental health research, it does not automatically translate into epistemic authority or structural influence. Further compounding these challenges, PWLE are frequently invited to, or rather placed into, LE research roles without adequate training, preparation, or institutional support necessary for success [20]. In our experience, many LE contributors are unaware of what type of role they are engaged in, and thus what expectations they have in research. Similarly, it seems as though many researchers are confused this as well. This is further illustrated by the inconsistent and inequitable remuneration practices that continue to exist: some PWLE are not compensated at all, while others receive payment that is either insufficient or disproportionately large. These trends reflect the absence of a standardized and scalable guide for lived experience involvement in research across institutional and national contexts that is universally accepted in the broader mental health research communities.

Hawke and colleagues [38] highlighted similar challenges in their critical reflections on embedding lived experience in research institutions, recommending a focus on well-planned, diversified initiatives, organizational commitment, research support mechanisms and risk mitigation strategies. Pointing to transparency and a break-down of different barriers, such as stigma, to guide LE involvement towards meaningful real-world benefits. Taken together it becomes evident that there is a desperate need for scientific consensus on *how* the broader academic community should engage PWLE in mental health research, while simultaneously embracing the dissensus that are a natural and important aspect in the growth of knowledge [39].

Undeniably, there is a need for a clear and coherent framework that delineates the different types of roles for PWLE in mental health research processes and institutions—one that both supports researchers, PWLE, and institutional administrations, while promoting a meaningful and sustained engagement.

Future work should aim at investigating how the framework may be used as a guide for financial procedures in academic institutions to enable successful embedment of lived experience in mental health research.

Nonetheless, this is, to our knowledge, the first theoretical paper to systematically delineate these distinct lived experience research roles, despite many individuals operating within them in practice [40]. By delineating the roles, the framework moves lived experience involvement from a general aspiration to a more operationalizable model that can inform recruitment, training, governance, funding and evaluation within mental health research institutions.

## Conclusion

In this paper, we have argued that meaningful lived experience involvement in mental health research requires more than unthoughtful compliance with funding requirements or general calls for action. Drawing on the historical foundations of lived experience advocacy and persistent challenges in research practice, we have demonstrated how ad hoc and poorly structured approaches risk involuntary tokenism and can undermine the legitimacy and value of lived experience expertise.

In response, we have outlined five meaningful lived experience research roles that people with lived experience can be engaged in. By naming and defining these roles, the framework provides a practical foundation for more thoughtful, meaningful, and ethically robust integration of lived experience in mental health research infrastructures. Crucially, the framework helps recognize LE involvement as a legitimate research practice that require the same standards of rigor, resourcing, and accountability as other forms of research expertise, shifting lived experience from a symbolic ideal to a structurally embedded component of mental health knowledge production.
